# Music Affects Rodents: A Systematic Review of Experimental Research

**DOI:** 10.3389/fnbeh.2018.00301

**Published:** 2018-12-14

**Authors:** A. Y. Rosalie Kühlmann, Aniek de Rooij, M. G. Myriam Hunink, Chris I. De Zeeuw, Johannes Jeekel

**Affiliations:** ^1^Department of Pediatric Surgery, Erasmus University Medical Center – Sophia Children's Hospital, Rotterdam, Netherlands; ^2^Department of Neuroscience, Erasmus University Medical Center, Rotterdam, Netherlands; ^3^Department of Radiology and Epidemiology, Erasmus University Medical Center, Rotterdam, Netherlands; ^4^Netherlands Institute for Neuroscience, Royal Netherlands Academy of Arts & Sciences, Amsterdam, Netherlands

**Keywords:** music, acoustic stimulation, animal models, neurogenesis, anxiety, brain, systematic review

## Abstract

**Background:** There is rapidly emerging interest in music interventions in healthcare. Music interventions are widely applicable, inexpensive, without side effects, and easy to use. It is not precisely known how they exert positive effects on health outcomes. Experimental studies in animal models might reveal more about the pathophysiological mechanisms of music interventions.

**Methods:** We performed a systematic review of experimental research in rodents. The electronic databases EMBASE, Medline(ovidSP), Web-Of-Science, PsycINFO, Cinahl, PubMed publisher, Cochrane, and Google scholar were searched for publications between January 1st 1960 and April 22nd 2017. Eligible were English–written, full-text publications on experimental research in rodents comparing music vs. a control situation. Outcomes were categorized in four domains: brain structure and neuro-chemistry; behavior; immunology; and physiology. Additionally, an overview was generated representing the effects of various types of music on outcomes. Bias in studies was assessed with the SYRCLE Risk of Bias tool. A meta-analysis was not feasible due to heterogeneous outcomes and lack of original outcome data.

**Results:** Forty-two studies were included. Music-exposed rodents showed statistically significant increases in neuro-chemistry, such as higher BDNF levels, as well as an enhanced propensity for neurogenesis and neuroplasticity. Furthermore, music exposure was linked with statistically significantly improved spatial and auditory learning, reduced anxiety-related behavior, and increased immune responses. Various statistically significant changes occurred in physiological parameters such as blood pressure and (para)sympathetic nerve activity following music interventions. The majority of studies investigated classical music interventions, but other types of music exerted positive effects on outcomes as well. The SYRCLE risk of bias assessment revealed unclear risk of bias in all studies.

**Conclusions:** Music interventions seem to improve brain structure and neuro-chemistry; behavior; immunology; and physiology in rodents. Further research is necessary to explore and optimize the effect of music interventions, and to evaluate its effects in humans.

## Introduction

There is growing interest in music interventions and music therapy in healthcare. Music interventions have a wide applicability, and the low cost, lack of side effects and ease of use make it an interesting intervention. Music interventions involve application of music in order to improve a clinical outcome, and can be administered recorded or live. They have been widely investigated in humans and can be linked to reduced depression levels in older people (Chan et al., 2011), to reduced disruptive behaviors and anxiety, and improved cognitive functioning in patients with dementia (Chang et al., 2015). A large number of studies have shown that music interventions alleviate anxiety and pain around medical procedures (Hole et al., 2015; Vetter et al., 2015) and surgical procedures (Kuhlmann et al., 2018). Music may have a beneficial effect on anxiety, systolic blood pressure, heart-rate, respiratory rate, quality of sleep, and pain in patients with coronary heart disease (Bradt et al., 2013), and might reduce blood-pressure in chronic hypertension (Kuhlmann et al., 2016). Lastly, music interventions appear to enhance immune function and to affect neuro-endocrine responses, such as a decrease in cortisol (Fancourt et al., 2014).

Music interventions are thought to not only exert their effects in humans by improving relaxation or providing distraction for a specific situation, but also to achieve specific physiological changes in the human body. The exact mechanism of action remains unknown. Music listening can influence a person's emotions and moods (Bennett and Lengacher, 2009; Mavridis, 2015) by activating specific pleasure areas in the limbic system, such as the nucleus accumbens, amygdala, and hippocampus (Blood and Zatorre, 2001; Menon and Levitin, 2005; Berridge and Kringelbach, 2015; Mavridis, 2015). These activations in turn may release neuropeptides, such as dopamine, and endogenous opioids (Blood and Zatorre, 2001; Mavridis, 2015). It cannot be excluded that such effects also occur in animals. Some studies in rodents indeed have shown that music exposure enhanced the expression of neuropeptides in the limbic system, which are known to be involved in pleasure and reward control (Sutoo and Akiyama, 2004; Feduccia and Duvauchelle, 2008; Tasset et al., 2012).

Moreover, several experimental studies in healthy rodents and in rodent disease models found similar effects as reported in humans, such as enhanced spatial ability (Xing et al., 2016c), improved neuroplasticity (Kirste et al., 2015), anxiety reduction (Escribano et al., 2014), blood pressure lowering (Sutoo and Akiyama, 2004), and increasing immune function (Uchiyama et al., 2012; Gao et al., 2016).

The outcomes of systematic experimental studies in animal models could be of value in understanding the working mechanisms of music interventions and extending clinical applicability of therapies. To answer the question whether music interventions exert effects on brain structure, neurochemistry, behavior, immunology, and physiology in rodents, we performed a systematic review of randomized experimental studies investigating music interventions in rodents compared to control situations.

## Methods

### Study Design

We performed a systematic review of the literature, and reported this following the PRISMA statement for transparent reporting of systematic reviews (Moher et al., 2009).

### Search Strategy and Data Sources

On April 22nd, 2017, a systematic literature search was performed in the electronical databases EMBASE, Medline(ovidSP), Web-Of-Science, PsycINFO, Cinahl, PubMed publisher, Cochrane, and Google scholar for publications that would be relevant to answer the research question (see Supplementary Material I Search Strategy). Titles and abstracts of citations were screened for relevance, and full texts of relevant citations were screened for relevance by two investigators (AK and AR) independently. In case of disagreement a third researcher (JJ) was consulted and consensus was negotiated.

### Participants, Interventions, Comparators

Studies meeting the following criteria were considered for inclusion: (1) experimental study performed in rats or mice; (2) investigating the effect of music interventions on neuronal processes, behavioral effects, endocrine and/or inflammatory responses, or physiological conditions; (3) comparing the effect of a music intervention with a comparator situation without music, referred to as “control;” (4) available full-text article; (5) written in English; (6) published after 1/1/1960. There were neither limitations to the type of music administered, the music had to contain melody, harmony, and rhythm (in case the intervention solely consisted of an auditory enrichment, such as white noise, the study was excluded); nor to the type of control condition. If study populations overlapped between studies, only the most extensively described study was included.

### Data Extraction and Data Analysis

The following study characteristics were collected in an Excel spreadsheet (Google Sheets, 2015): authors, year of publication, animal model characteristics (species, sex, age, number of animals, disease induced characteristics), music intervention (type, timing, duration, loudness), specific description of the music and genre, control condition (type, timing, duration, loudness), and performed tests. Study quality was assessed by two researchers (AK and AR) using the Systematic Review Center for Laboratory animal Experimentation (SYRCLE) Risk of Bias tool, which is the adapted version for animal studies of the Cochrane Risk of Bias tool (Hooijmans et al., 2014). Outcome measures were extracted by two persons separately and categorized into four areas: 1. brain structure and neuro-chemistry; 2. behavior; 3. immunology; and 4. physiology. Additionally, an overview was generated representing the effects of various types of music on outcomes. A meta-analysis was not performed due to the heterogeneity in outcomes and the lack of reporting original outcome data in reviewed studies.

## Results

### Study Selection and Characteristics

**Figure 1:**
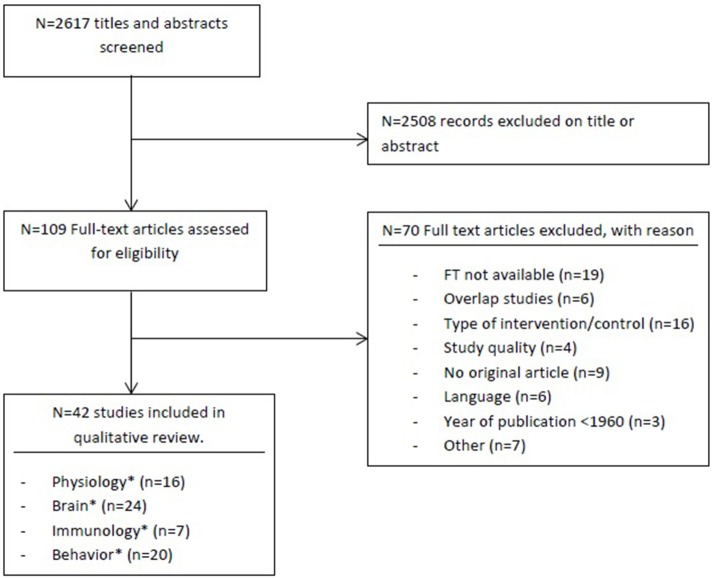
Flowchart. ^*^Some studies investigated outcomes on several areas.

**Figure 2:**
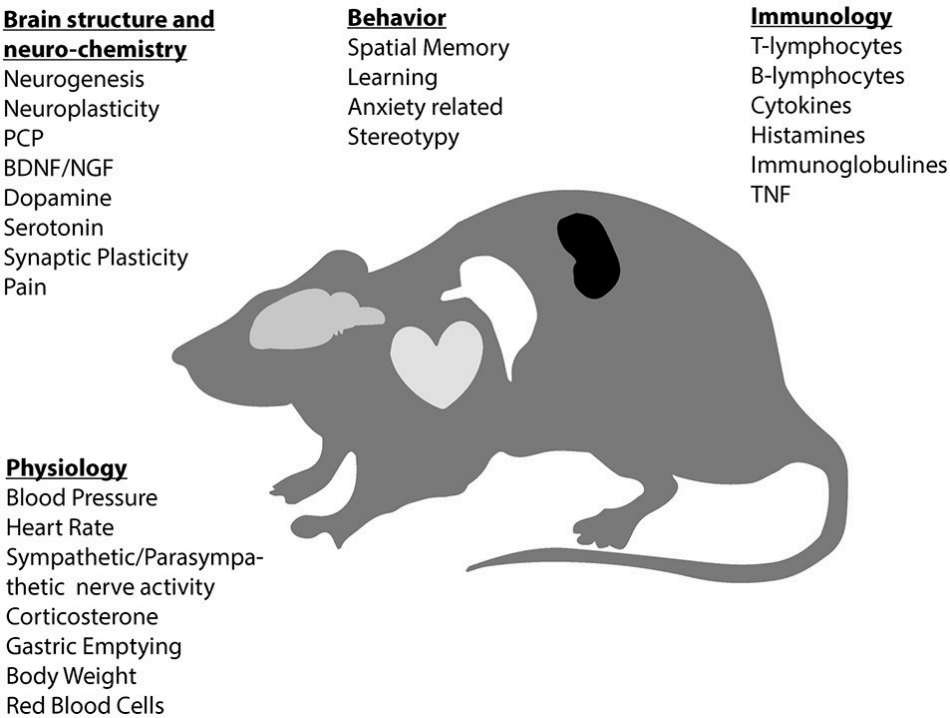
Music affects different domains in rodents. PCP, precursor cell proliferation; BDNF, brain derived neurotrophic factor; NGF, nerve growth factor; TNF, tumor necrosis factor.

The literature search resulted in 2,784 citations after removal of duplicates. Following eligibility assessment, 42 full-text articles were eligible for inclusion (see Figure 1). Detailed study characteristics are presented in Table 1. Figure 2 represents an overview of domains in rodents that seem affected by music. Thirty studies (71.4%) were in rats; twelve in mice. All studies investigated recorded music interventions played by loudspeaker. Control conditions were described as no music (17 studies, 40%); ambient noise (14 studies, 33%); white noise (5 studies, 13%); undisturbed situation (5 studies, 12%); and no stress (1 study, 2%). Twenty-eight studies (67%) involved several interventions/comparators (see Table 1).

**Table 1:** Study characteristics.

**Author**	**Year**	**Animal**	**Age**	***N*/group**	**Disease/Condition**	**Music Intervention**	**dB**	**Comparator**	**dB**	**Duration/frequency**	**Tests**
Gao	2016	Male Wistar rats	5–8 wk	10	Colorectal cancer bone cancer pain	Mozart K.448	60	No music	–	1 h/day for 2 weeks	Weight, tumor volume, pain, p38α, p38β
Jiang	2016	SAMP8 mice	7.5 mn	10	Alzheimer's disease	Musico-electro-acupuncture	–	**1. Electro acupuncture** 2. Alzheimer's control	–	20 min/day for 15 days	MWM test, brain glucose, amyloid-β frontal lobe
Lee	2016	Male SD rats	2 wk	8	Autism, valproic acid-induced	Comfortable classical music	65	Undisturbed		1 h/day from PND 15 to PND 28	SDAT; BDNF, TrkB, BrdU+ (HC)
Xing	2016c	SD rats	PND 1-98	5	–	Mozart K.448	70	**1. Ambient sound** 2. K.448 retrograde	65	12 h 8 p.m.−8 a.m.	MWM test
Xing	2016a	Male SD rats	PND 1-98	15	–	Mozart K.448	70	Ambient noise	65	12 h/ 8 a.m.to 8 p.m.	MWM test BDNF, TrkB
Xing	2016b	Male SD rats	adult	10	SE in TLE rats	Mozart K.448	70	**1. Ambient noise** 2. Control with saline (no SE)	75	2 h/day 8–10 p.m. day 1–34 after SE	MWM test swimming speed and distance
Cruz	2015	Albino Wistar rats	3–5 mn	10	Photoperiod (CD/SD/LD)	Mozart KV361	70	1. Ambient noise	50	24 h prior to and during tests	EPM test OPF test
Kim	2015	Male ICR mice	4 wk	5	Anaphylaxis induction	Korean Buk Music	70	**1. No music** 2. White noise	70	5 min	mortality, HIF-1α, VEGF, histamine, TNF-α, IL-1β
Kirste	2015	Female C57BL/6J mice	6–8 wk	10	–	Mozart K.448 (Transposed to 5–20 kHz)	70	**1. Ambient noise** 2. Silence 3. White noise 4. Pup calls	70	2 h/day in dark cycle, 3–7 days	BrdU+ cells, BrdU+/Sox2+ cells, cell differentiation
Sheikhi	2015	Wistar rat	prenatal day 2–20	6	–	Classical Music	60	No music	32	90 min 2/day	Corticosterone mother, neuroplasticity fetus
Escribano	2014	Female Wistar rat	3 mn	6	1. Normal 2. OVX/sham	Mozart K.448	65	**1. Ambient Noise** 2. White Noise	55	45 min before and during tests	EPM test LDT test
de Camargo	2013	Albino Wistar rats	3–5 mn	10	1. Simvastatin 2. Silence	Mozart KV361	70	1. Ambient noise	50	1 month music 5 h/day, then 24 h prior to/during tests	EPM test, OPF test, object recognition test
Kim	2013	SD Rats	new born	5	–	Comfortable music	65	**1. Control** 2. Noise	1. −2.95	1 h/day from day 15 pregnancy till delivery	neurogenesis: BrdU MC, SC Thickness MC, SC
Zhang	2013	Male Wistar Rat	–	5	LSSD (stress by bondage, diet irregularity)	Gong Tone	–	**1. No music** 2. Xiaoyoa Powder 3. Combined 4. No LSSD control	–	45 min	Gastrin, IgG, T-cell proliferation, macrophages
Marzban	2012	Male Wistar rat	new born	15	-	Mozart K.448	90	No music	–	6 h/night for 60 days	BDNF (HC)
Tasset	2012	Male Wistar rat	20 mn	5–6	1. normal 2. haloperidol blocking DA-system	Mozart K.448	65	No music	–	2^*^2 h/day over 4 days	brain dopamine (PFC, SN, MS) prolactin corticosterone
Uchiyama	2012	C57BL/6, CBA, BALB/c mice	8–12 wk			Opera	60	1. Mozart classical **2. No music**	40	24 h/day 6 days after Tx	*heart Tx*: survival,
								3. New Age 4. Different frequencies 5. Eardrum perforation			IL-4, IL-10, IL-3, TNF-γ *adoptive Tx*: splenocytes, CD4+, Foxp3, CD4+CD25+
Akiyama	2011	Male SHR	12 wk	10	SHR	Mozart K.205	70	**1. No music** 2. 4 kHz−16 kHz 3. 250 Hz−2 kHz 4. 32–125 Hz	35	10 h (12–22 h)	BP tail-cuff method
da Cruz	2011	Albino Wistar rats	3–5 mn	10	1. Saline 2. Simvastatin	Mozart KV361	70	1. Ambient noise	50	24 h prior to and during tests	EPM test OPF test
Amagdei	2010	female Wistar rat	new-born	10–16	1. PND1 sham surgery 2. PND1 callosotomy	Sham + 42 Mozart piano sonatas	70	**1. Sham** **+** **No music** 2. Callosotomy + music 3. Callosotomy + NM	–	12 h/night from PND2-PND32	T-maze MB
Li	2010	C57BL/6 wild type, BDNF^Met/Met^ and BDNF^+/−^ mice	adult, 2–3 mn	6–9	Anxiety by BDNF^Met/Met^ and BDNF^+/−^	Diverse Chinese Classical, Western Classical pieces	55	1. Ambient Noise **2. White Noise**	1.40 2. 55	6 h/day (18–24 h) for 3 wk	BDNF/TrkB mRNA and quantity (PFC, HC, amygdala), OPF, EPM test
Lu	2010	male Wistar rat	21 days	8	sensitized asthma, restraint stress (tube)	Asthma + Mozart K.448	55	1. Ambient Noise **2. Asthma** 3. Early asthma 4. Late asthma	50	6 h/day 18–24 h for 14 days from week 11	leukocytes, eosinophils, IL-4, IL-1β brain, corticosterone
Meng	2009	male C57BL/ 6J(B6) mice	28 days	20	-	Mozart K.448	55	Ambient noise	50	8 h/day 22–6 h 30 days	DNA microarray: gene expression changes FC/HC OPF test, MWM test, PA task
Nakamura	2009	male Wistar rats	-	5	-	Schumann Traumerei Op.15-7	50	**1. No stimulation** 2. White Noise	50	60 min by earphones	GVNA, c-Fos expression in AudC
Xu	2009	male SD rats	new-born	4	-	Mozart K.448	70	No music	55	12 h/d for 42 days starting PND 14	ASDT, SDDT, NR2B protein expression AudC
Erken	2008	female Wistar Albino rats	adult	7	-	Mozart pieces	70	**1. Control** 2. Rock Music 3. Noise	1.42 2. 70 3. 95	1 h/day for 14 days	RBC deformability RBC aggregation
Feduccia	2008	Male SD rats	adult	11/10	MDMA	Euphoric House	70	**1. White noise** 2. No added sound	70	During tests	CPP, NAcc DA, 5-HT
Lemmer	2008	Wistar-Kyoto rat (NR) and SHR	adult	5	Hypertension	Mozart No. 40	75	**1. Same but no music** 2. Ligeti rock music 3. White Noise	75	2 h	abdominal aorta sensor for SBP, DBP, HR
Angelucci	2007b	Male BALB/c mice	adult (40 days)	10		New Age Music (slow rhythm)	55	Ambient noise	50	6 h/day for 21 days 6–12 pm	HT BDNF, HT NGF, weight
Angelucci	2007a	Male BALB/c mice	adult (40 days)			New Age Music (slow rhythm)	55	Ambient noise	50	6 h/day for 21 days 6–12 pm	BDNF, NGF, PA task, weight
Chikahisa	2007	Female Slc:ddy mice	8 wk	13	1. OVX 2. Sham 3. Progesterone inhibitor	Mozart K.448	70	**1. Ambient Noise** 2. White noise	1.55 2. 70	30–45 min before and during test	OPF test, EPM test, LDT test, MB test
Nakamura	2007	Male Wistar rats	–	5	–	Schumann Traumerei	50	**1. White Noise** 2. Chopin Etude	50	60 min by earphone	arterial BP, RSNA, H3 receptor
Xu	2007	SD rats	–	5	–	**1. (Nightwish)** 2. (Nostalgy)	70	Control	<45	12 h/day from PND 14	GluR2 protein in AudC and ACC
Chikahisa	2006	Female Std:ddY mice	Prenatal 7 days, PND 1-68	7	–	Mozart K.448	70	1. Ambient Noise **2. White noise**	1.55 2. 70	Continuously played through dark period	Cross-maze test, BDNF, body weight, corticosterone
Kim	2006	Offspring SD rats	Prenatal	5	–	Music-applied	65	**1. Control** 2. Noise-applied	1. −2. 95	1 h/day from preND 15 until delivery	Radial-arm maze test PND21, BrdU cells (HC)
Kim	2004	Offspring SD rats	12 wk	5	-	Music-applied	65	**1. Control** 2. Noise-applied	1. −2. 95	1 h/day from preND 15 until delivery	TPH, 5-HT (DRN/MRN)
Sutoo	2004	Male SHR	12 wk	10	Hypertension	Mozart K. 205	70	Ambient noise	35	18–20 h daily	tail-cuff SBP, serum calcium, brain DA
Morton	2001	C57/BL6 mice	adult	9/10	METH	Bach BWV1041	95	1. The Prodigy 2. Loud WN **3. Ambient Noise**	1.95 2. 95 3. 65	3 h	seizures, locomotion, CPP, reactive gliosis
Nunez	2001	male BALB/c mice male SD rats	7–12 wk 2 mn	20 10	– W 256 carcinosarcoma	Herbert von Karajan Adagio	<40	**1. Unstimulated controls** 2. auditory stressor 3. auditory stressor and music	100	9 a.m.−2 p.m./8 days	Lymphocytes, T-cell proliferation, NK-cell activity, ACTH N tumor nodules, %metastasis
Rauscher	1998	rats	prenatal, PND 0-60	30	–	Mozart K.448	65	**1. White Noise** 2. Philip Glass	65	12 h nocturnal until PND 65	T-maze (working time, N errors)
McCarthy^*^	1992	male SD rats	-	6	–	Rock music (noise stress)	70	Usual environment	45	24 h	lymphocytes, IL1, superoxide anion, temp, activity counts
Bueno^*^	1988	male NMRI mice	-	6	Fasting	Acoustic stress (by music)	≤90	**1. No stress** 2. Cold stress	–	20 min	Gastric emptying

*Wk, weeks; mn, months; SD rat, Sprague Dawley rat; PND, post-natal day; NM, No Music; WN, White Noise; AS, Ambient Sound; MWM, Morris Water Maze; SDAT, Step Down Avoidance Task; EPM, elevated plus maze; MB, marble burying; LDT, light-dark transition test; OPF open field; PA task, passive avoidance task; ASDT, auditory signal-detection task; SDDT, sound duration discrimination task; CPP, center place preference SE, status epilepticus; TLE, temporal lobe epilepsy; SHR, spontaneously hypertensive rat; DA, dopamine; 5-HT, serotonin; TPH, tryptophan hydroxylase; HIF-1, hypoxia inducible factor-1; VEGF, vascular endothelial growth factor; METH, Methamphetamine; PP, place preference; NR, normotensive rat; (S)BP, (systolic) blood pressure; GVNA, gastric vagal nerve activity; RSNA, renal sympathetic nerve activity; LBD, Light-Dark Box transition test; LSSD, liver stagnation and spleen deficiency; FC, forebrain cortex; MC, motor cortex; SC, somatosensory cortex; HC, hippocampus; PFC, prefrontal cortex; FrC, frontal cortex; S, striatum; SN, striatal nucleus; MS, mesencephalon; CC, corpus callosum; AudC, auditory cortex; HT, hypothalamus; ACC, anterior cingulate cortex; DRN, dorsal raphe nuclei; MRN, median raphe nuclei; OVX, ovariectomized; Sham, sham operated; temp, temperature*.

* *studies in which music intervention was used as stressor*.

### Risk of Bias

All studies were assessed as unclear risk of bias according to the SYRCLE risk of bias tool (see Supplementary Material II SYRCLE Risk of Bias tool). Most studies did describe animal and housing characteristics, and reported some attrition bias. Information on sequence generation, allocation concealment, blinding of caregivers/investigators and random outcome assessment was barely reported.

### Findings: Music and Brain Structure and Neuro-Chemistry

Twenty-three studies investigated the effects of music on the neuro-anatomy of the brain (see Table 2) (Morton et al., 2001; Nunez et al., 2002; Kim et al., 2004, 2006, 2013; Sutoo and Akiyama, 2004; Chikahisa et al., 2006; Angelucci et al., 2007a,b; Xu et al., 2007, 2009; Feduccia and Duvauchelle, 2008; Meng et al., 2009; Li et al., 2010; Marzban, 2012; Tasset et al., 2012; Kirste et al., 2015; Sheikhi and Saboory, 2015; Gao et al., 2016; Jiang et al., 2016; Lee et al., 2016; Xing et al., 2016a,c), such as neurogenesis and neuroplasticity as measured by precursor cell proliferation by bromodeoxyuridine (BrdU) labeled cells, levels of brain derived neurotrophic factor (BDNF) expression, and nerve growth factor (NGF); levels of dopamine and serotonine; seizures; expression of amyloid-β; and effects on neuronal pain pathways.

**Table 2:** Brain outcomes.

**Author**	**Year**	**Outcome**	**Result music**	**Result comparator**	***P*-value**	**Music; comparator**
Xing	2016	BNDF/TrkB	↑		<0.05	Mozart K.448; AS
Xing	2016	BDNF/ TrkB				Mozart K448; AN
		*dCA3&dDG*	↑		<0.05	
		*dCA1*	=		n.s.	
Lee	2016	BDNF/TrkB	↑		<0.05	Classical music; NM
		BrdU + cells	↑		<0.05	
Marzban	2012	BDNF	94.60 ± 6.22	86.30 ± 2.26	<0.01	Mozart K.448; NM
Li	2010	BDNF				Chinese/Western Classical; WN
		*PFC/ HC/ Amygdala*	↑/↑/↑		<0.05	
		BDNF/TrkB-mRNA				
		*PFC*	↑		<0.05	
		*HC/Amygdala*	↑/↑		<0.01	
Angelucci	2007a	BDNF				New Age Music; NM
		*HC/FrC/S*	↑/=/=		<0.05/ns/ns	
		NGF				
		*HC/FrC/S*	=/=/=		ns/ns/ns	
Angelucci	2007b	BDNF *HT*	↑		<0.01	New Age Music; NM
		NGF *HT*	↓		<0.05	
Chikahisa	2006	BDNF				Mozart K.448; WN
		*Cortex*	↓		<0.05	
		*HC/cerebellum*	=		n.s.	
		TrkB				
		*Cortex*	↑		<0.05	
		*HC*	=		n.s.	
Sheikhi	2015	Density PC	7.17 ± 0.6	5.5 ± 0.43	<0.05	Classical music; NM
Kirste	2015	BrdU+ cells *N*	↑		<0.01	Mozart K.448; AN
		BrdU+/Sox2+ *N*	↑		<0.01	
		Diff cells	=		n.s.	
Kim	2013	BrdU MC *N cells*	486.79 ± 47.21	371.56 ± 29.29	<0.05	Comfortable music; Control
		BrdU SC *N cells*	926.26 ± 93.44	660.72 ± 58.90	<0.05	
		Thickness MC (mm)	1.204 ± 0.034	1.277 ± 0.034	n.s.	
		Thickness SC (mm)	1.241 ± 0.035	1.305 ± 0.023	n.s.	
Kim	2006	BrdU cells (HC) *N cells*				Music; Control
		*CA1*	3229.59 ± 119.04	2352.00 ± 111.40	<0.05	
		*CA2/CA3*	1393.70 ± 57.66	868.00 ± 40.50	<0.05	
		*Dentate gyrus*	2055.72 ± 124.39	2367.28 ± 138.25	n.s.	
Tasset	2011	Dopamine (ng/g)				Mozart K.448; NM
		*PFC*	96.00 ± 3.75	73.01 ± 2.02	<0.01	
		*SN*	69.70 ± 2.08	60.15 ± 2.84	<0.05	
		*MS*	71.60 ± 1.75	58.59 ± 2.20	<0.001	
Sutoo	2004	Dopamine (FI)				Mozart K.205; NM
		*lateral neostriatum*	5.31 ± 0.16	4.51 ± 0.21	<0.01	
		*MC, SC, N Acc*	=		n.s.	
Feduccia	2008	Dopamine N.acc.	↑		<0.05	House Music; WN
		5-HT	↑		<0.05	
Kim	2004	5-HT				Music; Control
		*DRN*	109.09 ± 10.77	159.15 ± 5.47	<0.05	
		*MRN*	37.93 ± 3.23	53.16 ± 2.18	<0.05	
		TPH				
		*DRN*	153.94 ± 7.81	184.32 ± 9.92	<0.05	
		*MRN*	42.50 ± 2.57	65.58 ± 3.10	<0.05	
Meng	2009	Gene expression				Mozart K.448; AN
		FrC *(N genes)*	454	-		
		HC *(N genes)*	437	-		
Xu	2009	NR2B protein expression	163.00 ± 18.9	88.65 ± 22.7	0.046	Mozart K448; NM
		AudC				
Nakamura	2009	c-Fos expression AudC	↑		<0.05	Traumerei; NS
Xu	2007	GluR2 expression				Nightwish; Control
		*AudC (nmol/mg)*	1499.47 ± 114.55	860.31 ± 64.31	<0.05	
		*ACC (nmol/mg)*	2809.37 ± 191.83	1490.00 ± 90.63	<0.01	
Morton	2001	Seizures (% mice)	75.0%	38.7%	<0.01	Bach + METH; Silence + METH
		Reactive gliosis	↑		<0.05	
Nunez	2001	ACTH	=		n.s.	Adagio; UC
Jiang	2016	Brain glucose metabolism	↑		<0.05	MEA; EA
		Amyloid- β accumulation	↓		<0.05	
Gao	2016	p38α expression	35.4 ± 3.7	71.2 ±3.9	0.014	Mozart K.448; NM
		p38β expression	40.2 ± 3.5	68.5 ± 3.3	0.018	
		foot withdrawal (*time s)*	10.4 ± 3.2	28.7± 6.2	0.011	
		heat pain threshold *(time s)*	49.3 ± 5.7	27.8 ± 4.3	0.031	
		free walking pain *(time s)*	2.5 ± 0.3	3.6 ± 0.6	0.033	

*The signs “↑/↓/ = ” mean higher/ lower/ equal compared to control when no specific original data were presented. NM, no music; NS, no stimulation; WN, white noise; AN, ambient noise; UC, unstimulated control; (M)EA, (musico) elektro acupuncture; METH, Methamphetamine; 5-HT, serotonin; TPH, tryptophan hydroxylase; DA, dopamine; DRN, dorsal raphe nuclei; MRN, median raphe nuclei; FI, fluorescence intensity; MC, motor cortex; SC, somatosensory cortex; HC, hippocampus; N Acc, nucleus accumbens; dCA1/3/DG, hippocampal region CA1/3/dental gyrus; PC, parietal cortex; PFC, prefrontal cortex; FrC, frontal cortex; S, striatum; SN, striatal nucleus; MS, mesencephalon; CC, corpus callosum; HT, hypothalamus; AudC, auditory cortex; ACC, anterior cingulate cortex*.

All four studies that investigated effects of music on levels of BrdU-cells found increased levels compared to a control condition (Kim et al., 2006, 2013; Kirste et al., 2015; Lee et al., 2016). Prenatal music increased the number of cells in the motor cortex and somatosensory cortex (Kim et al., 2013) as well as in the hippocampal CA1, CA2, and CA3 regions, but not in the dental gyrus (Kim et al., 2006). Moreover, the brain cells of rat fetuses exposed to music were morphologically more complex than those of rat fetuses not exposed to music (Sheikhi and Saboory, 2015). Music statistically significantly increased levels of BDNF compared to comparator situations in seven out of eight studies (Angelucci et al., 2007a,b; Li et al., 2010; Marzban, 2012; Lee et al., 2016; Xing et al., 2016a,c)—specifically in cells of the dorsal CA3 region of the hippocampus (HC), the dentate gyrus (Xing et al., 2016a), the prefrontal cortex, amygdala, and hypothalamus (Angelucci et al., 2007a,b; Li et al., 2010); whereas the NGF level was not altered in cells of the CA1 region (Xing et al., 2016a). One study found a decrease of BDNF in the cortex and no change in the HC and the cerebellum compared to comparator conditions (Chikahisa et al., 2006). One study found that music decreased nerve growth factor in the hypothalamus (Angelucci et al., 2007b), while it had no impact on the HC, frontal cortex or striatum (Angelucci et al., 2007a). In the same two studies, BDNF levels were elevated in both the HC and the hypothalamus.

The three studies investigating effects of music on dopamine levels in the brain (Sutoo and Akiyama, 2004; Feduccia and Duvauchelle, 2008; Tasset et al., 2012) found either an increase of dopamine in the nucleus accumbens (Feduccia and Duvauchelle, 2008); in the prefrontal cortex, mesencephalon and the striatum (Tasset et al., 2012); or no differences in dopamine in the motor cortex, somatosensory cortex, or nucleus accumbens (Sutoo and Akiyama, 2004). Music prevented the decrease of dopamine after administration of a D2-receptor antagonist in rats (Tasset et al., 2012). In another study, music up-regulated the expression of dopamine-related genes in mice (Meng et al., 2009). Effects of music on serotonin levels were investigated in two studies (Kim et al., 2004; Feduccia and Duvauchelle, 2008): prenatal music decreased serotonin synthesis in the dorsal and median raphe nuclei in the offsprings (Kim et al., 2004); but it increased serotonin in the nucleus accumbens after administration of methamphetamine (Feduccia and Duvauchelle, 2008).

When methamphetamine was injected in mice, exposure to either rave or classical music increased the numbers of seizures and deaths, suggesting increased methamphetamine toxicity (Morton et al., 2001). Rats exposed to music showed a significant increase in the expression of the NMDA receptor NR2B protein in their auditory cortex (Xu et al., 2009). Similarly, the expression of another glutamate receptor subunit which can be involved in synaptic plasticity, GluR2, was also significantly increased in the auditory cortex following music exposure, suggesting induced plasticity in the auditory system (Xu et al., 2007).

In a mouse model of Alzheimer's disease, addition of music to electro-acupuncture treatment statistically significantly improved the glucose metabolism level in the mice's brains, while the expression of amyloid-β, which is normally accumulated in Alzheimer's disease, was decreased (Jiang et al., 2016). Lastly, the one study examining effects of music on cancer bone pain found less pain intensity as well as decreased expression of p38α and p38β in the dorsal ganglia, which are involved in processing chronic neuropathic, inflammatory, and cancer pains (Gao et al., 2016).

### Findings: Music and Behavior

Twenty-one studies investigated the effects of music on behavioral outcomes (see Table 3) (Rauscher et al., 1998; Morton et al., 2001; Chikahisa et al., 2006, 2007; Kim et al., 2006; Angelucci et al., 2007a; Feduccia and Duvauchelle, 2008; Meng et al., 2009; Xu et al., 2009; Amagdei et al., 2010; Li et al., 2010; da Cruz et al., 2011; de Camargo et al., 2013; Escribano et al., 2014; Cruz et al., 2015; Jiang et al., 2016; Lee et al., 2016; Xing et al., 2016a,b,c; Yazdani et al., 2016)—specifically learning abilities, anxiety-related behavior and stereotypic behavior as investigated by behavioral tests explained in Supplementary Material III.

**Table 3:** Behavior outcomes.

**Author**	**Year**	**Outcome**	**Result music**	**Result comparator**	***P*-value**	**Music; comparator**
Xing	2016	*MWM-test*				Mozart K.448; AS
		TET	↓		<0.05	
		TTQ	↑		<0.01	
Xing	2016	*MWM-test*				Mozart K.448; AN
		TET	↓		<0.05	
		TTQ	↑		<0.05	
		Swimming speed	=		n.s.	
		Swimming distance	=		n.s.	
		Learning rate	↑		<0.05	
Xing	2016	*MWM-test*				Mozart K.448; AN
		TET	↓		<0.01	
		TTQ	↑		<0.05	
Jiang	2016	*MWM-test*				MEA; EA
		TET	↓		<0.05	
		TTQ	↑		<0.05	
		Swimming speed	↑		<0.05	
Lee	2016	SDAT	↑		<0.05	Classical music; Undisturbed
Amagdei	2010	*T-maze*				Mozart; NM
		Alteration				
		Performance	↑		<0.01	
		Response latency	=		n.s.	
		MB test	=		n.s.	
Cruz	2015	*EPM-test*				Mozart KV361; AN
		TTS	↑		<0.01	
		EOA	↑		n.s.	
		Grooming time	↑		<0.01	
		Rearing time	↑		<0.01	
Escribano	2014	*EPM-test*				Mozart K.448; AN
		TTS	↑		<0.01	
		EOA	↑		<0.01	
		*LBD-test*				
		TSLS	↑		<0.01	
		LBLS	↓		<0.01	
de Camargo	2013	*EPM test*				Mozart KV361; AN
		TTS	↑		<0.05	
		EOA	↑		<0.01	
		*OPF test*				
		Locomotion	↑		<0.01	
		TTI	↓		<0.05	
		ORT	=		n.s.	
da Cruz	2011	*EPM test*				Mozart KV361; AN
		TTS	↑		<0.05	
		EOA	=		n.s.	
		*OPF test*				
		Locomotion	=		n.s.	
		TTI	=		n.s.	
Li	2010	*OPF-test*				Chinese & Western Classical; WN
		Locomotion	=		n.s.	
		TTC	↑		<0.01	
		*EPM-test*				
		TTS	↑		<0.05	
		EOA	↑		<0.01	
Meng	2009	*OPF test*	=		n.s.	Mozart K.448; AN
		Escape latency	↓		<0.05	
		TTQ	↑		<0.05	
		*PA-task*				
		Escape latency	↑		<0.01	
Xu	2009	*ASDT*				Mozart K.448; NM
		Correct licking	=		0.097	
		Rate				
		Performance index	↑		0.005	
		SDDT	↑		<0.01	
Feduccia	2008	CPP	=		n.s.	House Music; WN
Chikahisa	2007	*EPM test*				Mozart K.448; AN
		TTS	↑		<0.01	
		EOA	↑		<0.01	
		DOA	↑		<0.01	
		*OPF test*				
		TDO	=		n.s.	
		TTC	↑		<0.05	
		*LDT test*				
		TSLS	↑		<0.05	
		LBLS	↓		<0.05	
		*MB-test*	↓		<0.05	
Angelucci	2007a	*PA task*				New Age music; AN
		LBLS	↑		<0.05	
		N trials to learn	↓		<0.05	
Chikahisa	2006	*X-maze test*				Mozart K.448; WN
		Running time	=		n.s.	
		Errors (N)	↓		<0.01	
Kim	2006	*Radial-arm maze test*				Music; Control
		Total time to complete	63.00 ± 7.73	110.88 ± 14.42	<0.05	
		N correct choice	6.90 ± 0.23	6.44 ± 0.29	n.s.	
		N errors	3.20 ± 0.85	5.55 ± 1.00	n.s.	
Morton	2001	CPP	↑		<0.01	Bach + METH; Silence + METH
		Stereotypy	↑		–	
Rauscher	1998	Working time	34.72	44.29	<0.05	Mozart K.448; WN
		N errors	2.0	3.35	<0.01	WN

*The signs “↑/↓/ = ” mean higher/ lower/ equal compared to control. NM, no music; WN, white noise; AN, ambient noise; AS, ambient sound; (M)EA, (musico) elektro acupuncture; MWM, Morris Water Maze; TET, Total Escape Time; TTQ, Time in Target Quadrant; SDAT, Step-down avoidance task; MB, marble burying; EPM, elevated-plus-maze; TTS, total time spent in open arms; EOA, entries in open arms; DOA, distance in open arms; LBT, Light-Dark Transition; TSLS, time spent light side; LBLS, latency before entering light side; OPF, open field; TDO, total distance in OPF test; TTC, total time center; TTI, total time immobile; ORT, object recognition test; PA-task, passive avoidance task; ASDT auditory signal detection test; SDDT, sound duration discrimination task; CPP, center place preference; X-maze, cross-maze*.

Music interventions enhanced learning abilities of rodents, specifically those involved with spatial learning (Rauscher et al., 1998; Chikahisa et al., 2006; Kim et al., 2006; Xu et al., 2009; Amagdei et al., 2010; Jiang et al., 2016; Lee et al., 2016; Xing et al., 2016a,b,c; Yazdani et al., 2016). Moreover, music statistically significantly decreased anxiety-related behavior in seven out of nine studies (Angelucci et al., 2007a; Chikahisa et al., 2007; Meng et al., 2009; Li et al., 2010; de Camargo et al., 2013; Escribano et al., 2014; Cruz et al., 2015); the remaining two studies found no differences between music and comparator groups (Amagdei et al., 2010; da Cruz et al., 2011). The anxiety-decreasing effect of music diminished after ovariectomy and was restored by progesterone (Chikahisa et al., 2007; Escribano et al., 2014). Music seemed to enhance anxiolytic effects of simvastatin (da Cruz et al., 2011; de Camargo et al., 2013). Influence of music on stereotypic behavior was investigated in two studies; music enhanced stereotypic behavior after administration of methamphetamine, but not of saline (Morton et al., 2001; Feduccia and Duvauchelle, 2008).

### Findings: Music and Immunology

Seven studies investigated the effects of music on immunological outcomes (see Table 4) (McCarthy et al., 1992; Nunez et al., 2002; Lu et al., 2010; Uchiyama et al., 2012; Zhang et al., 2013; Kim et al., 2015; Gao et al., 2016), such as specific and non-specific immunity; cytokines and histamines; anaphylaxis; tumor growth; and post-transplantation immunity.

**Table 4:** Immunologic outcomes.

**Author**	**Year**	**Outcome**	**Result music**	**Result comparator**	***P*-value**	**Music; comparator**
Gao	2016	Tumor volume	32.6 ± 12.2	114.3 ± 24.7	0.008	Mozart K.448; NM
Kim	2015	Mortality *(%)*	44.33 ± 14.01	77.77 ± 9.62	<0.05	Korean Buk; NM
		TNF-α	0.60 ± 0.15	1.44 ± 0.17	<0.05	
		Histamine	41.53 ± 1.53	52.72 ± 2.93	<0.05	
		IL-1β	1.41 ± 0.43	1.37 ± 0.12	n.s.	
		HIF-1	1.07 ± 0.33	1.80 ± 0.39	<0.05	
		VEGF	0.116 ± 0.009	0.172 ± 0.008	<0.05	
Zhang	2013	Gastrin	=		n.s.	Gong Tone; NM
		IgG (μg/ml)	64.18 ± 1.89	42.80 ± 8.98	<0.01	
		T cell (*SI*)	2.30 ± 0.19	2.03 ± 0.06	<0.01	
		Phagocytosis (*OD*)	0.36 ± 0.08	0.18 ± 0.07	<0.01	
						
Uchiyama	2012	*heart Tx:*				Opera; NM
		MST *(days)*	26.5	7	<0.001	
		Foxp3CD4+CD25+	↑		<0.001	
		IL-4	↑		<0.01	
		IL-10	↑		<0.05	
		IL-3	↓		<0.05	
		IFN-γ	↓		<0.05	
		*adoptive Tx:*				
		Splenocytes MST *(days)*	36	10	<0.01	
		CD4+ MST *(days)*	68	8	<0.001	
		CD4+CD25+ MST *(days)*	>100	8	<0.005	
Lu	2010	IL-4 (ng/ml)	1.10 ± 0.17	0.73 ± 0.12	-	Asthma + Mozart K.448; Asthma
		IL-1β brain (ng/ml)	0.082 ± 0.003	0.080 ± 0.004	n.s.	
		Leukocytes lung	↓		<0.05	
		Eosinophils	↓		<0.05	
Nunez	2001	Lymphocytes	↑		<0.05	Adagio; UC
		T-cell proliferation	↑		<0.01	
		NK-cell activity	↑		<0.01	
		Tumor nodules *(N)*	=		n.s.	
		Area of metastasis *(%)*	↓		<0.05	
McCarthy*	1992	Lymphocytes *(N cells)*	4413 ± 766	4392 ± 1046	<0.0001	Rock music; UE
		Superoxide anion	2.0 ± 1.5	4.9 ± 9	<0.01	
		IL-1	↓		<0.05	

The signs “↑/↓/ = ” mean higher/ lower/ equal compared to control. NM, no music; UE, usual environment; UC, unstimulated control; MST, median survival time; SI, stimulation index; OD, optical density. ^*^studies in which music intervention was used as stressor.

Music exposure enhanced the numbers of lymphocytes and natural killer cells as well as the levels of T-cell proliferation and phagocytosis (McCarthy et al., 1992; Nunez et al., 2002; Zhang et al., 2013). Noise stress induced by loud rock music resulted in statistically significantly decreased production of superoxide anion and IL-1, suggestive of deprived leucocyte function (McCarthy et al., 1992). Gong tone music up-regulated plasma-cells and proliferation of T-cells in rats with deprived spleen function (Zhang et al., 2013), and music exposure significantly decreased the number of eosinophils and increased cytokine levels in asthmatic rats compared to controls (Lu et al., 2010). Mice exposed to Korean Buk music showed a statistically significantly decreased production of cytokines and histamines as well as statistically significantly lower mortality from anaphylactic shock (Kim et al., 2015). Decreased tumor volume and decreased area of metastasis was found in the presence of music (Nunez et al., 2002; Gao et al., 2016). Rodents exposed to opera or classical music had statistically significantly prolonged survival after heart transplantation. Moreover, adoptive transfer of splenocytes and T-cells from music-exposed rodents into naïve recipients was associated with prolonged survival of these recipients (Uchiyama et al., 2012).

### Findings: Music and Physiology

Sixteen studies investigated effects of music on physiological outcomes in rodents (see Table 5) (Bueno and Gue, 1988; McCarthy et al., 1992; Sutoo and Akiyama, 2004; Chikahisa et al., 2006; Kim et al., 2006; Angelucci et al., 2007a,b; Nakamura et al., 2007, 2009; Erken et al., 2008; Lemmer, 2008; Lu et al., 2010; Akiyama and Sutoo, 2011; Tasset et al., 2012; Sheikhi and Saboory, 2015; Gao et al., 2016), including blood pressure and heart rate; sympathetic and parasympathetic nerve activity; corticosterone levels; body weight and digestion; and red blood cell activity.

**Table 5:** Physiologic outcomes.

**Author**	**Year**	**Outcome**	**Result Music**	**Result Comparator**	**P-value**	**Music; Comparator**
Akiyama	2011	BP (mmHg)	↓ 16–28		<0.01	Mozart K.205; NM
Sutoo	2004	BP (mmHg)	↓ 13–24		<0.05	Mozart K.205; NM
		Serum calcium	↑ 5–6%		<0.05	
Lemmer	2008	*NR*				Mozart No.40; own control (cross-over!)
		SBP (mmHg)	=		n.s.	
		DBP (mmHg)	=		n.s.	
		HR (b/min)	=		n.s.	
		*SHR*				
		SBP (mmHg)	=		n.s.	
		DBP (mmHg)	=		n.s.	
		HR (b/min)	↓		<0.035	
Nakamura	2009	GVNA (% baseline)	↑ 154.9 ± 18.5		<0.05	Traumerei; NS
Nakamura	2007	MAP (% baseline)	↓ 87.9 ± 6.1		<0.05	Traumerei; WN
		RSNA (% baseline)	↓ 32.8 ± 10.6		<0.05	
Erken	2008	RBCD	↑		<0.05	Classical; Control
		RBCA	↓		<0.01	
Lu	2010	Corticosterone	6.47 ± 0.10	7.11 ± 0.16	<0.05	Asthma + Mozart K.448; Asthma
Tasset	2012	Corticosterone	15.18 ± 0.62	19.27 ± 2.14	<0.01	Mozart K.448; NM
		Prolactin	19.90 ± 0.76	28.48 ± 1.75	<0.01	
Sheikhi	2015	Corticosterone	29.53 ± 1.43	37.01 ± 2.58	0.02	Classical music; NM
		Body weight	=		n.s.	
Chikahisa	2006	Corticosterone	=		n.s.	Mozart K.448; WN; NM
		Body weight	=		n.s.	
Angelucci	2007a	Body weight	=		n.s.	New age music; AN
Angelucci	2007b	Body weight	=		n.s.	New age music; AN
Kim	2006	Body weight	=			Music-applied; Control
McCarthy*	1992	Temperature	↑		–	Rock music; NM
		Activity counts	10.3 ± 3.2	8.1 ± 5.1	<0.001	
Bueno*	1988	GE (% total meal)	62.8 ± 15.5	42.5 ± 6.5	≤0.05	Acoustic stress; Control
Gao	2016	Weight (gram)	−4.9 ±1.2	−10.5 ± 1.3	0.012	Mozart K.448; NM
		Feed efficiency ratio	62.3 ± 5.8	35.4 ± 6.2	0.026	

The signs “↑/↓/ = ” mean higher/ lower/ equal compared to control when no specific original data were presented. NM, no music; NS, no stimulation; WN, white noise; AN, ambient noise; BP, blood pressure; HR, heart rate; GVNA, gastric vagal nerve activity; RSNA, renal sympathetic nerve activity; RBCD, red blood cell deformity; RBCA, red blood cell aggregation; GE, Gastric Emptying. ^*^studies in which music intervention was used as stressor.

Four studies investigated effects of classical string music on blood pressure (Sutoo and Akiyama, 2004; Nakamura et al., 2007; Lemmer, 2008; Akiyama and Sutoo, 2011); of which one also investigated effects on heart rate (Lemmer, 2008). A statistically significantly decrease in blood pressure was noted in three out of four studies. High-frequency music was more effective in decreasing blood pressure than was low-frequency music, with an absent effect at the lowest frequencies (Akiyama and Sutoo, 2011). Sympathetic nerve activity and blood pressure decreased after music exposure (Nakamura et al., 2007) while parasympathetic nerve activity increased (Nakamura et al., 2009). Three out of four studies found significantly decreased corticosterone levels after music interventions (Lu et al., 2010; Tasset et al., 2012; Sheikhi and Saboory, 2015). While exposure to music was followed by a statistically significantly decrease of blood corticosterone in pregnant rats (Sheikhi and Saboory, 2015), this phenomenon was not seen in the offspring upon pre- and postnatal daily exposure to music (Chikahisa et al., 2006). Classical music exposure decreased red blood cell functioning (Erken et al., 2008). Acoustic stress by rock music increased gastric emptying, however administration of anti-corticotropic releasing factor prevented this (Bueno and Gue, 1988). Of six studies that evaluated the effect of music on body weight (Chikahisa et al., 2006; Kim et al., 2006; Angelucci et al., 2007a,b; Sheikhi and Saboory, 2015; Gao et al., 2016), one found statistically significantly weight reduction (Gao et al., 2016).

### Types of Music

Overall, studies used a wide range of music interventions. Classical music was the most investigated intervention (29 studies, 70.7%; of which 14 studies used Mozart's sonata for two pianos, K.448). Table 6 represents an overview of the genres of music interventions and their effects on outcomes. Most studies investigating classical music found positive effects on outcomes regarding brain structure and neurochemistry, and on outcomes regarding behavior such as spatial memory or anxiety. Positive effects on physiological outcomes were also seen and suggested decreased sympathetic activity. Majority of these classical music studies investigated Mozart music, specifically Mozart K.448. Retrograde versions of this music piece had negative effects on spatial memory, this effect was also present when rodents heard the music for the first time. Furthermore, blood pressure decreasing effects were seen in high frequency music, while these effects were not present in low frequency music.

**Table 6:** Music genres and their effect on outcomes.

**Type music**	**Specification**	**N**	**+/=/−**	**Outcome (specification)**
Classical	Mozart *K.448*	14	+	↓ anxiety (Chikahisa et al., 2007; Escribano et al., 2014) ↑ spatial memory/learning (Rauscher et al., 1998; Chikahisa et al., 2006; Meng et al., 2009; Xu et al., 2009; Xing et al., 2016a,b,c) ↓ tumor gene expression; ↑ pain threshold (Gao et al., 2016) ↑ neuroplasticity (Marzban, 2012; Xing et al., 2016c), in hippocampus (Xing et al., 2016b), in cortex (Chikahisa et al., 2006), in auditory cortex (Xu et al., 2009) ↑ neurogenesis (Kirste et al., 2015), in motor cortex/somatosensory cortex/hippocampus (Kim et al., 2006, 2013) ↑ dopamine prefrontal cortex/striatal nucleus/mesencephalon (Tasset et al., 2012) ↑ immune function, decreased innate immunity (Lu et al., 2010) ↓ tumor volume; ↓weight loss (Gao et al., 2016) ↓ corticosterone (Lu et al., 2010) ↓ corticosterone; ↓prolactin (Tasset et al., 2012)
			=	equal neuroplasticity hippocampus (Chikahisa et al., 2006) equal neurogenesis dental gyrus (Kim et al., 2006) equal corticosterone; equal body weight (Chikahisa et al., 2006) equal physical performance (Chikahisa et al., 2006; Xing et al., 2016c) gene expression result not specified (Meng et al., 2009)
	Mozart *K.448 (retrograde)*	1	−	↓ spatial memory (Xing et al., 2016a)
	Mozart *KV361*	3	+	↓ anxiety (de Camargo et al., 2013; Cruz et al., 2015)
			=	equal anxiety (da Cruz et al., 2011) equal learning (de Camargo et al., 2013)
	Mozart *n.40*	1	+	↓ heart rate (Lemmer, 2008)
			=	equal blood pressure (Lemmer, 2008)
	Mozart *K.205*	1	+	↑ dopamine striatum, ↓ blood pressure (Sutoo and Akiyama, 2004)
			=	equal dopamine motor cortex/somatosensory cortex/nucleus accumbens (Sutoo and Akiyama, 2004)
	Mozart *K.205 high frequency*	1	+	↓ blood pressure (Akiyama and Sutoo, 2011)
	Mozart *K.205 low frequency*	1	=	equal blood pressure (Akiyama and Sutoo, 2011)
	Mozart	3	+	↑ immune function and prolonged graft survival (Uchiyama et al., 2012) ↓ heart rate and erythrocyte functioning (Erken et al., 2008) ↑ learning (Amagdei et al., 2010)
			=	equal blood pressure (Erken et al., 2008) equal anxiety (Amagdei et al., 2010)
	Schumann *Traumerei*	2	+	↓ blood pressure, ↓sympathetic activity (Nakamura et al., 2007) ↑ parasympathetic activity, ↑ neuroplasticity (Nakamura et al., 2009)
	Bach *BWV1041*	1	=	equal anxiety (Morton et al., 2001)
			−	↑ percentage seizures and ↑ reactive gliosis (Morton et al., 2001)
	Herbert von Karajan *Adagio*	1	+	↑ immunity and ↓ tumor area (Nunez et al., 2002)
			=	equal number tumor nodules (Nunez et al., 2002)
	Chopin *Etude*	1	=	equal blood pressure, equal sympathetic activity (Nakamura et al., 2009)
	Philip Glass (minimalistic)	1	=	equal spatial memory (Rauscher et al., 1998)
	Classical (Chinese/Western)	1	+	↓ anxiety; ↑ neuroplasticity hippocampus, prefrontal cortex, amygdala (Li et al., 2010)
	Classical (not specified)	3	+	↑ learning, neuroplasticity, neurogenesis (Lee et al., 2016) ↓ corticosterone; ↑density parietal cortex (Sheikhi and Saboory, 2015)
			=	equal body weight (Sheikhi and Saboory, 2015)
Opera	Opera	1	+	↑ immune function and graft survival (Uchiyama et al., 2012)
New Age	New Age	3	+	↑ learning, ↓ anxiety (Angelucci et al., 2007a,b) ↑ neuroplasticity hippocampus (Angelucci et al., 2007a), hypothalamus (Angelucci et al., 2007b)
			=	equal immune function and graft survival (Uchiyama et al., 2012) equal bodyweight (Angelucci et al., 2007a,b) equal neuroplasticity frontal cortex/striatum; equal neurogenesis (Angelucci et al., 2007a) ↓ nerve growth factor (Angelucci et al., 2007b)
Cultural	Korean Buk	1	+	↓mortality and ↓ activity cytokines and histamines (Kim et al., 2015)
	Gong tone	1	+	↑ cellular immunity (Zhang et al., 2013)
			=	equal production gastrin (Zhang et al., 2013)
Up beat	Euphoric house	1	+	↑ dopamine nucleus accumbens; ↑ serotonin (Feduccia and Duvauchelle, 2008)
			=	equal anxiety (Feduccia and Duvauchelle, 2008)
	Prodigy *Electronic*	1	+	↓ anxiety (Morton et al., 2001)
			=	equal *n* of seizures and reactive gliosis, equal stereotypic behavior (Morton et al., 2001)
	Ligeti *Rock music*	2	+	↓ blood pressure (Erken et al., 2008)
			=	equal heart rate, equal erythrocyte functioning (Erken et al., 2008)
			−	↑ immunology response, ↑ activity immune system (McCarthy et al., 1992)
Music (not specified)	Music	3	+	↑ learning (Kim et al., 2006) ↓ serotonin (5-HT) Raphe nuclei prenatally (Kim et al., 2004) ↑ spatial memory; ↑ physical performance; ↑ brain glucose metabolism (Jiang et al., 2016)
			=	equal learning; equal body weight (Kim et al., 2006)
	Comfortable	1	+	↑ neurogenesis motor cortex/somatosensory cortex/mesencephalon (Kim et al., 2013)
	Nightwish	1	+	↑ synaptic plasticity (Xu et al., 2007)
	Nostalgy	1	=	equal synaptic plasticity (Xu et al., 2007)
	Acoustic stress	1	−	↑ gastric emptying (Bueno and Gue, 1988)

*The signs “+/ = /−” mean “positive/ equal/ negative” effect on outcome compared to control. Some studies investigated several types of music and several outcomes*.

Other types of music showed variable effects. New age music increased neuroplasticity in one study compared to the control group, but did not affect neurogenesis or immunologic outcomes. Anxiety and learning were however improved.

Cultural music was investigated in two studies that both found positive results on immunologic functioning. Up-beat music also showed variable results. Rock music did not positively affect any outcomes, whereas electronic house music did decrease anxiety, and euphoric house music did increase dopamine and serotonin levels.

Studies that used non-specified music interventions found positive results as well, such as increased spatial memory, increased learning and increased physical performance. Studies that used music as acoustic stressor did not find positive results on outcomes.

Seven of 42 studies compared several types of music interventions (Rauscher et al., 1998; Morton et al., 2001; Xu et al., 2007; Erken et al., 2008; Lemmer, 2008; Nakamura et al., 2009; Uchiyama et al., 2012) and allowed direct comparison of music on the outcomes due to the equality of the study conditions. A statistically significant increase in functional brain activity and plasticity was found after exposure to Nightwish music, this effect was not present after exposure to Nostalgy music (Xu et al., 2007), however absence of specific description of these two music pieces inhibited a formal comparison between the types of music. Electronic music temporarily decreased anxiety after supplementation of methamphetamine whereas classical Bach music did not (Morton et al., 2001), and classical Mozart music statistically significantly increased spatial memory compared to minimalistic classical music by Philip Glass (Rauscher et al., 1998). There was no difference in neurologic outcomes after exposure to loud classical music by Bach, or to loud modern electronic music by The Prodigy (Morton et al., 2001). Both classical and opera music significantly improved immune function and graft survival, whereas New Age music did not had any significant effect on these parameters (Uchiyama et al., 2012). Ligeti rock music, but not Mozart music, resulted in a long-lasting blood pressure decreasing effect, Mozart music on the other hand was significantly effective in reducing heart rate (Erken et al., 2008). Both classical and rock music affected the erythrocyte response to stress with higher degree of significance in the classical music group (Erken et al., 2008). Exposure to Schumann's Traumerei resulted in decreased sympathetic activity, but exposure to an Etude by Chopin did not (Nakamura et al., 2009).

## Discussion

### Summary of Findings

The results of this systematic review indicate that music exposure can exert positive effects on rodents' neurological, behavioral, immunological, and physiological outcomes. These results are broadly consistent with studies in humans that found that music exposure can positively affect brain structure and chemistry (Johansson, 2011; Yeh et al., 2015), behavioral read-outs (Chan et al., 2011; Thoma et al., 2013; Baird and Samson, 2015; Hole et al., 2015; Vetter et al., 2015), immunological responses (Conrad et al., 2007; Fancourt et al., 2014), and physiological parameters (Bekiroglu et al., 2013; Hole et al., 2015).

Music exposure increased rodents' spatial memory and learning in all studies that examined it. Music seems to specifically affect spatial memory, the one study examining non-spatial memory did not find any differences between the music and control situations (de Camargo et al., 2013). Exposure to music decreased anxiety in all included studies. Both spatial memory and anxiety might be affected by the level of BDNF. Low levels of BDNF have been associated with anxiety and aggressive behavior in mice (Akbarian et al., 2002; Li et al., 2010) and with anxiety and depression in humans (Martinowich et al., 2007; Brunoni et al., 2008). This protein is involved in synaptic plasticity, learning, and memory areas of the brain, such as the hypothalamus and hippocampus, and regulates neuronal structure and function (Mizuno et al., 2000; Chikahisa et al., 2006; Angelucci et al., 2007b; Marzban, 2012). In most studies examining, BDNF levels were elevated following exposure to music, and this might explain the reduced anxiety. The improved behavioral performance on spatial memory tasks and anxiety tests after music interventions is likely to be, at least in part, the effect of increased levels of BDNF. This finding suggests that music exposure has the potential to improve neuroplasticity and neurogenesis in the brain. This could be of value in the treatment of psychological disorders or acquired brain injuries and should be further explored (Kim et al., 2006; Kirste et al., 2015; Xing et al., 2016a).

Furthermore, music exposure possibly counteracts the adverse effects of stress and thereby enhances the immune function. Music interventions were associated with increased functions of cellular and humoral immunity, increased phagocytosis and increased production of lymphocytes and immunoglobulins (Zhang et al., 2013). In rodent cancer models, music exposure was associated with lower tumor volume and smaller area of metastasis (Nunez et al., 2002; Gao et al., 2016). Regarding allergic reactions such as anaphylaxis, however, the immune system seemed tempered in the presence of music—with lower production of cytokines and histamines and thereby less mortality (Kim et al., 2015). Remarkably, this effect of music also manifests itself in survival after transplantation. Enhanced production of anti-inflammatory cytokines and regulatory T-cells restrained the immune-system in the presence of music and thereby significantly lengthened the survival times of transplants (Uchiyama et al., 2012). Comparable effects of music on immunological and neurochemical functions have also been reported in humans (Bartlett et al., 1993; Stefano et al., 2004; Fancourt et al., 2014). This promising result should be further investigated.

Physiological effects induced by music are commonly explained by attenuation of autonomic function by stress reduction. Stress affects the hypothalamic-pituitary-adrenal axis and the sympathetic nervous system in humans and animals alike. Stress reduction causes the sympathetic activity to shift to more parasympathetic activity, resulting in lower heart rate and blood pressure. The blood pressure-reducing effect of music has extensively been described in humans (Bekiroglu et al., 2013; Kuhlmann et al., 2016), and it may hold for rodents as well. Corticosterone, the rodent's equivalent of human cortisol, is involved in regulating stress-responses and is an important biomarker for stress. Music interventions were associated with reduced corticosterone levels in several animal models (Chikahisa et al., 2006; Tasset et al., 2012). Comparable effects of music on cortisol have been reported in humans (Leardi et al., 2007; Koelsch et al., 2011). In addition, the blood pressure reduction might be induced by autonomic regulation via sympathetic suppression by histaminergic receptors (Nakamura et al., 2007), or on calcium level regulation via the calmodulin system (Sutoo and Akiyama, 2004; Xu et al., 2007). An increase of calcium ions enhances dopamine synthesis, and increased dopamine levels in turn may inhibit sympathetic activity via specific D2 receptors and thus reduce blood pressure (Sutoo and Akiyama, 1997, 2004; Tasset et al., 2012). Increased calcium influx in the brain might be due to excitatory impulses, also represented by enhanced synaptic transmission (Xu et al., 2007). Enhanced synaptic transmission can result in improved learning and memory functions, and boosts the formation of neural networks during brain development (Ozawa et al., 1998; Dumas, 2005; Xu et al., 2007).

### Working Mechanisms of Music

The specific mechanisms by which music exerts its effects are unknown. It seems that at least the auditory pathway must be intact, as effects of music were not seen after lesions of the eardrum (Uchiyama et al., 2012), cochlea, auditory cortex, and suprachiasmatic nucleus (Nakamura et al., 2007). As for the type of music, most of the studies in this review used classical music, with a preference for music composed by Mozart. This may be described to what is known as the Mozart-effect (Rauscher et al., 1998), which implies an enhanced effect on spatial memory by listening to music composed by Mozart. As the findings of replication studies are inconsistent (Newman et al., 1995; Rideout and Laubach, 1996; Steele et al., 1999) a firm conclusion on the Mozart-effect cannot be drawn. Most of the 12 studies investigating other types of music, including folk music such as Korean Buk music (Kim et al., 2015) or Gong tone music (Zhang et al., 2013) found statistically significant results as well, suggesting there is more to music than just the classical component. Different physiological effects were observed when playing different musical pieces, even when the music was in the same genre (Lemmer, 2008) or from roughly the same classical style (Nakamura et al., 2009). More complex classical music seemed of more value to spatial memory than minimalistic classical music did (Rauscher et al., 1998). One study compared tonal classical music of Mozart with the avant-garde classical music of Ligeti, the latter characterized by micro tonality and dissonant harmonies that could be subjectively described as unsettling (Lemmer, 2008). Both pieces yielded opposite effects, suggesting that musical factors like tone, harmony, or melody are all important in exerting effects. Effects of specific intervals, rhythm, and melodies can also be seen in another study in which rats' spatial performance was negatively affected with reversed versions of the music, while original versions positively affected performance compared to controls (Xing et al., 2016c). In this study rhythm appeared to be a crucial element (Xing et al., 2016c). In other studies, rhythm also appeared to be important to achieve positive effects. Cultural music involving Gong tone or Buk instruments, both characterized by strong rhythmic patterns, induced positive effects on immunology (Zhang et al., 2013; Kim et al., 2015). These specific components of music triggering pathophysiological mechanisms warrant further investigation.

While low-frequency music altered or even abolished effects of music in rodents, higher frequency notes resulted in better responses (Akiyama and Sutoo, 2011; Uchiyama et al., 2012). Hearing abilities of rodents differ from those of humans, varying from 500 Hz to 64 kHz in rats and 2 kHz to 80 kHz in mice to 20 Hz to 20 kHz in humans (Heffner and Heffner, 2007; Rosen and Howell, 2013), which could explain improvement of results with higher frequency notes. No significant differences on neurologic outcomes were found between exposure to classical or rave music after methamphetamine injection (Morton et al., 2001), however, music was played loudly and this might have been so stressful that it suppressed any effects. In addition, impaired immune function was seen after exposure to loud rock music (McCarthy et al., 1992), again suggesting that music volume might affect any outcomes.

## Limitations

The outcome of this systematic review faces several limitations. The sample sizes of the included studies were generally small. Additionally, we found a substantial unclear risk of bias (see Supplementary Material II) with the SYRCLE risk of bias tool (Hooijmans et al., 2014). Music interventions were heterogeneous and sometimes sparsely described. Furthermore, studies were performed in different populations and also with various types of control situations. Not every study considered the day-night cycle of rodents. When interpreting the results of this review, one should be aware of these limitations.

## Conclusion

This systematic review finds music interventions to improve outcomes of brain structure and neuro-chemistry, behavior, immunology, and physiology in rodents. These results support application of music as intervention in many healthcare areas. Future studies in both rodents and humans could look more into matters of musical complexity, rhythm, and pitch as well as the frequency with which music interventions are offered.

## Author Contributions

AK, AdR, and JJ conceived the study idea. AK and AdR coordinated the systematic review. AK and AdR screened abstracts and full texts. AK and AdR wrote the first draft of the manuscript and judged risk of bias in the studies. AK, AdR, MH, CDZ, and JJ interpreted the data. AK, AdR, MH, CDZ, and JJ critically revised the manuscript. AK, AdR, MH, CDZ, and JJ had full access to all of the data in the study and can take responsibility for the integrity of the data and the accuracy of the data analysis.

### Conflict of Interest Statement

CDZ was supported by ZonMw, NWO-ALW, ERC-Adv and ERC-PoC outside of the submitted work. MH was supported by ZonMw, EIBIR, ESR, and CUP outside of the submitted work. The remaining authors declare that the research was conducted in the absence of any commercial or financial relationships that could be construed as a potential conflict of interest.

## Abbreviations

SYRCLE: Systematic Review Centre for Laboratory animal Experimentation
BrdU: bromodeoxyuridine
BDNF: brain derived neurotrophic factor
HC: hippocampus
NMDA: N-methyl-D-aspartate
NR2B: subunit NMDA receptor
GluR2: glutamate receptor.
