# Supplementary material for: Music Affects Rodents: A Systematic Review of Experimental Research
Source: Front Behav Neurosci. 2018 Dec 14;12:301. doi: 10.3389/fnbeh.2018.00301 (PMC6302112; doi:10.3389/fnbeh.2018.00301)
Supplement: Supplementary file 1 [file Data_Sheet_1.docx]

**Supplementary Material I: Data Sheet I search strategy**

| Embase.com | 1037 | 1016 |
| --- | --- | --- |
| Medline (ovidSP) | 547 | 91 |
| Web-of-science | 807 | 473 |
| Scopus | 1007 | 220 |
| Cochrane | 19 | 1 |
| PsycINFO (ovidSP) | 962 | 646 |
| Cinahl | 88 | 43 |
| Google scholar | 200 | 127 |
| **Total** | **4667** | **2617** |

**Embase.com**

(music/de OR 'music therapy'/de OR (music OR musical OR musicotherap*):ab,ti) AND ([animals]/lim OR (animal* OR rat OR rats OR mouse OR mice OR murine):ab,ti)

**Medline (ovidSP)**

(music/ OR "music therapy"/ OR (music OR musical OR musicotherap*).ab,ti.) AND ((exp animals/ NOT humans/) OR (animal* OR rat OR rats OR mouse OR mice OR murine).ab,ti.)

**PsycINFO (ovidSP)**

(music/ OR "music therapy"/ OR (music OR musical OR musicotherap*).ab,ti.) AND ((exp animals/ NOT humans/) OR (animal* OR rat OR rats OR mouse OR mice OR murine).ab,ti.)

**Cochrane**

((music OR musical OR musicotherap*):ab,ti) AND ((animal* OR rat OR rats OR mouse OR mice OR murine):ab,ti)

**Web-of-science**

TS=(((music OR musical OR musicotherap*)) AND ((animal* OR rat OR rats OR mouse OR mice OR murine)))

**Scopus**

TITLE-ABS-KEY(((music OR musical OR musicotherap*)) AND ((animal* OR rat OR rats OR mouse OR mice OR murine))) AND doctype(ar)

**Cinahl**

(MH music OR MH "music therapy" OR (music OR musical OR musicotherap*)) AND ((MH animals+ NOT MH humans+) OR (animal* OR rat OR rats OR mouse OR mice OR murine))

**Google scholar**

Music|musical animal|animals|rat|rats|mouse|mice|murine
