# Supplementary material for: Music Affects Rodents: A Systematic Review of Experimental Research
Source: Front Behav Neurosci. 2018 Dec 14;12:301. doi: 10.3389/fnbeh.2018.00301 (PMC6302112; doi:10.3389/fnbeh.2018.00301)
Supplement: Supplementary file 2 [file Data_Sheet_2.docx]

**Supplementary Material II: SYRCLE Risk of Bias Table**

| **Author, year** | **Selection bias: Baseline Characteristics** | **Selection bias: sequence generation and allocation concealment** | **Performance bias: Random Housing** | **Performance bias: blinding of caregivers and/or investigators** | **Detection bias: outcome assessment** | **Attrition bias: incomplete outcome data** | **Reporting bias; selective outcome reporting** | **Other: other possible sources of bias.** |
| --- | --- | --- | --- | --- | --- | --- | --- | --- |
| **Gao 2016** | Low | Unclear | Low | Unclear | Unclear | Unclear | Unclear | Unclear |
| **Jiang 2016** | Low | Unclear | Unclear | Unclear | Unclear | Unclear | Unclear | Unclear |
| **Lee 2016** | Low | Unclear | Unclear | Unclear | Unclear | Unclear | Unclear | Unclear |
| **Xing 2016 (1)** | Low | Unclear | Low | Unclear | Unclear | Unclear | Unclear | Unclear |
| **Xing 2016 (2)** | Low | Unclear | Low | Unclear | Unclear | Unclear | Unclear | Unclear |
| **Xing 2016 (3)** | Low | Unclear | Low | Unclear | Unclear | Unclear | Unclear | Unclear |
| **Cruz 2015** | Low | Unclear | Low | Unclear | Unclear | Unclear | Unclear | Unclear |
| **Kim 2015** | Unclear | Unclear | Unclear | Unclear | Unclear | Unclear | Unclear | Unclear |
| **Kirste 2015** | Low | Unclear | Unclear | Unclear | Unclear | Unclear | Unclear | Unclear |
| **Sheikhi 2015** | Low | Unclear | Unclear | Unclear | Unclear | Unclear | Unclear | Unclear |
| **Escribano 2014** | Low | Unclear | Low | Unclear | Unclear | Unclear | Unclear | Unclear |
| **de Camargo**  **2013** | Low | Unclear | Unclear | Unclear | Unclear | Unclear | Unclear | Unclear |
| **Kim 2013** | Low | Unclear | Low | Unclear | Unclear | Unclear | Unclear | Unclear |
| **Zhang 2013** | Low | Unclear | Low | Unclear | Unclear | Unclear | Unclear | Unclear |
| **Marzban 2012** | Unclear | Unclear | Low | Unclear | Unclear | Unclear | Unclear | Unclear |
| **Tasset 2012** | Low | Unclear | Unclear | Unclear | Unclear | Unclear | Unclear | Unclear |
| **Uchiyama 2012** | Low | Unclear | Low | Unclear | Unclear | Unclear | Unclear | Unclear |
| **Akiyama 2011** | Low | Unclear | Unclear | Unclear | Unclear | Unclear | Unclear | Unclear |
| **da Cruz 2015** | Low | Unclear | Unclear | Unclear | Unclear | Unclear | Unclear | Unclear |
| **Amagdei 2010** | Low | Unclear | Low | Unclear | Low | Unclear | Unclear | Unclear |
| **Li 2010** | Low | Unclear | Low | Unclear | Low | Unclear | Unclear | Unclear |
| **Lu 2010** | Low | Unclear | Low | Unclear | Unclear | Unclear | Unclear | Unclear |
| **Meng 2009** | Low | Unclear | Low | Unclear | Low | Unclear | Unclear | Unclear |
| **Nakamura 2009** | Low | Unclear | Low | Unclear | Unclear | Unclear | Unclear | Unclear |
| **Xu 2009** | Low | Unclear | Unclear | Unclear | Unclear | Unclear | Unclear | Unclear |
| **Erken 2008** | Low | Unclear | Unclear | Unclear | Unclear | Unclear | Unclear | Unclear |
| **Feduccia 2008** | Low | Unclear | Unclear | Unclear | Unclear | Unclear | Unclear | Unlcear |
| **Lemmer 2008** | Low | Unclear | Low | Unclear | Unclear | Unclear | Unclear | Unclear |
| **Angelucci 2007 (1)** | Low | Unclear | Unclear | Unclear | Unclear | Unclear | Unclear | Unclear |
| **Angelucci 2007 (2)** | Low | Unclear | Unclear | Unclear | Unclear | Unclear | Unclear | Unclear |
| **Chikahisa 2007** | Low | Unclear | Unclear | Unclear | Unclear | Unclear | Unclear | Unclear |
| **Nakamura 2007** | Low | Unclear | Unclear | Unclear | Unclear | Unclear | Unclear | Unclear |
| **Xu 2007** | Unclear | Unclear | Unclear | Unclear | Unclear | Unclear | Unclear | Unclear |
| **Chikahisa 2006** | Low | Unclear | Unclear | Unclear | Unclear | Unclear | Unclear | Unclear |
| **Kim 2006** | Low | Unclear | Low | Unclear | Unclear | Unclear | Unclear | Unclear |
| **Kim 2004** | Low | Unclear | Low | Unclear | Unclear | Unclear | Unclear | Unclear |
| **Sutoo 2004** | Unclear | Unclear | Unclear | Unclear | Unclear | Unclear | Unclear | Unclear |
| **Morton 2001** | Low | Unclear | Unclear | Unclear | Low | Unclear | Unclear | Unclear |
| **Nunez 2001** | Low | Unclear | Low | Unclear | Unclear | Unclear | Unclear | Unclear |
| **Rauscher 1998** | Unclear | Unclear | Low | Unclear | Low | Unclear | Unclear | Unclear |
| **McCarthy 1992** | Unclear | Unclear | Unclear | Unclear | Unclear | Unclear | Unclear | Unclear |
| **Bueno 1998** | Unclear | Unclear | Unclear | Unclear | Unclear | Unclear | Unclear | Unclear |
