# Supplementary material for: Music Affects Rodents: A Systematic Review of Experimental Research
Source: Front Behav Neurosci. 2018 Dec 14;12:301. doi: 10.3389/fnbeh.2018.00301 (PMC6302112; doi:10.3389/fnbeh.2018.00301)
Supplement: Supplementary file 3 [file Data_Sheet_3.docx]

**Supplementary Material III: Data Sheet 2 explanatory box behavioral tests**

T-Maze: Measures natural tendency of animal to enter an alternate arm if they know they previously visited the other arm (spatial memory). Animal is centrally placed in T-maze with one open arm which can be explored, the other arm is closed. Subsequently the animal is replaced in the start area, but now both arms are open. During training days, animals are given 5 or 10 trials. During test day, number of alterations and response latency are recorded.

Cross-Maze: A cross-maze consists of a center platform that extends four arms in a cross-formation and an arm leading to a start box (closed after starting). Each arm is equipped with two water nozzles (real and dummy) with four different marks. During learning tests, only one arm provided water, and when a rodent drank a water from the drinkable nozzle, the drinkable nozzle moved to the next arm in clockwise order. In testing days, trial ended when rodents drank water for two rounds (two times per arm) or after 10 minutes. Total running time in the maze and total number of errors (entering an arm without drinkable nozzle) were recorded.

Morris Water Maze: Pool with opaque water and a hidden platform below surface in a target quadrant. During training days, rats learned to find and climb the platform (spatial memory). During test day, the platform was removed and rats explored the pool for 60s. Total time spent in target quadrant was measured.

Elevated Plus Maze: Rodents are placed in center of an apparatus consisting of two opposing open arms and enclosed arms. Behavior is monitored for 5 minutes. Number of entries and time spent in open arms is recorded (anxiety).

Radial Arm Maze: Apparatus consists of a central octagonal plate and eight radiating arms. End of the arms contained small water basin. Rodents were allowed to explore for water and drink for 5 minutes. At test day, time spent for seeking and drinking water at the end of arms was counted, and test ended after rodent found water in all eight arms or when 5 minutes passed. Re-entering in a previously visited arm was an error, number of correct choices before error was also counted (spatial memory).

Marble Burying: Animal is placed in a cage with 5cm deep wood chip bedding with equally divided marbles on it. Number of marbles buried in a period of time is measured (stress behavior).

Light Dark Transition: Chamber with equally divided light compartment and dark compartment (separated by wall with opening). Rodent placed in dark compartment, and latency to go out into the light side for the first time was recorded as well as amount of time spent in each compartment for period of time (anxiety).

Open Field: Registration of locomotor behavior of rodents in an open top box for certain time period. The total distance and time spent in the central and peripheral area are recorded, as well as time spent immobile (anxiety).

­Passive Avoidance task: On training day, rodent is placed in a light compartment, allowing to explore for time period. After certain time, a door is raised and rodent is allowed to explore the dark compartment. When dark compartment is entered with four paws (latency-time), door is closed and rodents receive a foot-shock. Retest session was a single trial without foot-shock, total time until animal entered other compartment was measured.

Conditioned Place Preference: Apparatus consists of two compartments (black and white) separated by removable wall. After 5 minutes habituation, duration of time spent in each compartment was registered over time period.

Step Down Avoidance Task: Evaluation of short memory. Rodents rest on a platform for 2 minutes. When stepping down the platform, a foot-shock is given for 2 seconds. 2 days after training session latency in step-down-avoidance-task was determined as time interval between the moment when rats first stepped down and when they placed all four paws on the ground.

Auditory Signal Detection Task: rodents are required to respond to sound stimuli by licking the water spout. Number of correct licks, missed licks and false licks is recorded. Each correct lick was rewarded with water. Correct licking rate is calculated.

Sound Duration Discrimination Task: Subsequently presentation of two sounds of different duration. Licking the spout during presentation of one of two sounds was rewarded with water, while licking in response to the other sound was not rewarded.
